# Identifying the risk: a prospective cohort study examining postpartum haemorrhage in a regional Australian health service

**DOI:** 10.1186/s12884-018-1852-8

**Published:** 2018-06-07

**Authors:** Lauren Kearney, Mary Kynn, Rachel Reed, Lisa Davenport, Jeanine Young, Keppel Schafer

**Affiliations:** 1 Women and Families Service Group, Sunshine Coast Hospital and Health Service, Sunshine Coast University Hospital, 6 Doherty St, Birtinya, Qld, Birtinya, 4575 Australia; 2 University of the Sunshine Coast, Locked Bag 4, Maroochydore DC, Qld 4558 Australia; 3 Sunshine Coast Hospital and Health Service, Birtinya, Queensland, Australia

**Keywords:** Postpartum haemorrhage, Third stage labour management, Active management, Expectant management, Blood loss measures, Estimated blood loss, Gravimetric

## Abstract

**Background:**

In industrialised countries the incidence of postpartum haemorrhage (PPH) is increasing, for which exact etiology is not well understood. Studies have relied upon retrospective data with estimated blood loss as the primary outcome, known to be underestimated by clinicians. This study aimed to explore variables associated with PPH in a cohort of women birthing vaginally in coastal Queensland, Australia, using the gravimetric method to measure blood loss.

**Methods:**

Women were prospectively recruited to participate using an opt-out consent process. Maternal demographics; pregnancy history; model of care; mode of birth; third stage management practices; antenatal, intrapartum and immediate postpartum complications; gravimetric and estimated blood loss; and haematological laboratory data, were collected via a pre-designed data collection instrument. Descriptive statistics were used for demographic, intrapartum and birthing practices. A General Linear Model was used for multivariate analysis to examine relationship between gravimetric blood loss and demographic, birthing practices and intrapartum variables. The primary outcome was a postpartum haemorrhage (blood loss > 500 ml).

**Results:**

522 singleton births were included in the analysis. Maternal mean age was 29 years; 58% were multiparous. Most participants received active (291, 55.7%) or modified active management of third stage (191, 36.6%). Of 451 births with valid gravimetric blood loss recorded, 35% (*n* = 159) recorded a loss of 500 ml or more and 111 (70%) of these were recorded as PPH. Gravimetric blood loss was strongly correlated with estimated blood loss (*r* = 0.88; *p* < 0.001). On average, the estimated blood loss was lower than the gravimetric blood loss, about 78% of the measured value. High neonatal weight, perineal injury, complications during labour, separation of mother and baby, and observation of a gush of blood were associated with PPH. Nulliparity, labour induction and augmentation, syntocinon use were not associated with PPH.

**Conclusions:**

In contrast to previous study findings, nulliparity, labour induction and augmentation were not associated with PPH. Estimation of blood loss was relatively accurate in comparison to gravimetric assessment; raising questions about routine gravimetric assessment of blood loss following uncomplicated births. Further research is required to investigate type and speed of blood loss associated with PPH.

## Background

The third-stage of labour is known as the time from the birth of the baby to the complete expulsion of the placenta and membranes [1]. The most common complication of the third stage of labour is postpartum haemorrhage (PPH) [2]. Both within Australia, and the international context, a primary PPH is defined as blood loss greater than 500 ml within the first 24 h following a vaginal birth [3–5]. Globally, PPH is the leading cause of maternal mortality [6], and a significant cause of morbidity. During 1994 less than 5% of women birthing experienced a PPH in Australia, and this increased to just under 7% in 2005 [7]. Associated factors which have also increased during this time are maternal age, caesarean birth, multiple pregnancies and induced labours [7]. Between 2008 and 2012, 12 (11%) of the Australian maternal deaths were attributable to obstetric haemorrhage [8], with the incidence of severe PPH (> 1000 ml) in Queensland doubling between 2001 (*n* = 1229; 2%) and 2016 (*n* = 1491; 3%) [3]. Internationally, the incidence is also on the rise, especially in industrialised countries, but reasons for this are not clear [9].

Despite the routine use of active management of the third stage of labour (current recommendation aimed at reducing PPH), PPH rates in high resource countries continue to rise [7]. Active management of the third stage of labour involves three components: administration of a utertonic, cord clamping (previously this was immediate, however now is often deferred to allow for placental transfusion in line with emerging evidence) [10, 11], and application of controlled cord traction to deliver the placenta [2]. Expectant management allows for physiological delivery of the placenta by maternal effort and gravity, and is usually offered in industrialised countries upon an individual woman’s choice and the preferred absence of risk factors for PPH.

Whilst active management is known to reduce the risk of PPH for many women [2], other contributing factors have also been associated with the upward trend in PPH throughout industrialised countries, including: increases in maternal age (≥35 years old) [12, 13], caesarean birth [13, 14], multiple pregnancies [15], labour inductions [14] and augmentation of labour [14, 16]. As exact aetiology is unknown, all women should be monitored for excessive blood loss following childbirth. Other international studies, based on retrospective data, have indicated that nulliparity [9, 17], episiotomy [17], retained placenta [17], high neonatal body weight [17], low antenatal haemoglobin [17], physiological management of the third stage in mixed-risk women [2], prior caesarean section [14], placenta previa [9], transverse lie [14, 18], and labour induction and augmentation [9] are the major independent risk factors for PPH. In addition, the increasing use of synthetic oxytocin to induce and augment labour has been significantly associated with severe PPH due to uterine atony [16]. However, changes in risk factors, such as increasing maternal age, accounted for only 5.6% of the increase in severe PPH in Kramer and colleagues’ [14] large study (*n* = 8.5 million), and further studies are warranted to understand the changing trends.

A substantial limitation of the retrospective data from which the previously mentioned studies are based upon, is the inaccuracy of health care professionals’ estimation of blood loss [19, 20]; the *primary outcome* of these studies. Standard practice within birthing units (including the study setting) is to estimate postpartum blood loss and commence more accurate measurement in dishes, or through weighing, once the midwife is concerned. Whilst this has been accepted practice, there are concerns regarding the inaccuracy of this practice and the speed at which a postpartum haemorrhage can progress. Glover [21] conducted a small study (*n* = 21) examining the accuracy of obstetricians, obstetric registrars and registered midwives in estimating blood loss through simulated ‘blood’ on pads, draw sheets and absorbent pads. Consistently the health care professionals underestimated the blood loss, and this underestimation increased proportionate to the blood loss. Similarly, another more recent Australian study (*n* = 88), aimed to determine the accuracy of estimating blood loss using simulated examples [22], by Registered Midwives. Participants were more accurate in their estimations with smaller loss and if it was contained in a measurable dish, yet wide variation in estimation was apparent, for example at a measured 600 ml loss, estimations varied from 50 ml through to 400 ml. Internationally, studies have confirmed this underestimation. Al Kadri and colleagues [19] conducted a prospective cohort study in Saudi Arabia and found a significant difference between the gravimetric (weighed) calculated blood loss and health care providers’ estimation, with a tendency to underestimate the loss by around 30%. This underestimation was not affected by the seniority of the health care provider. Overestimation has also been found to occur. Yoong and colleagues’ study conducted in London found that smaller volumes of estimated blood loss were especially inaccurate, being overestimated by up to 540% [23]. This study also found that level of experience did not appear to have a confounding effect on accuracy. Gravimetric measurement of postpartum blood loss has been shown to be a much more accurate method of quantifying blood loss than estimation alone [24]; while previous studies have relied on estimation alone to determine which risk factors are then associated with a PPH. Gravimetric measurement involves a clear quantification of blood loss through use of weighing blood soaked pads, linen and/or drapes [19].

Another important variable which has not been well accounted for in studies predicting risk for PPH are the changes which have evolved specifically within the traditional components of the actively managed third-stage of labour. The most recent Cochrane review comparing active with physiological management of the third stage of labour [2] determined that for women of ‘mixed risk’, active management reduced the risk of severe PPH by 34% . However, modifications have been made to active management in recent years (such as delayed cord clamping), and no studies to date have assessed the impact these modifications have had on PPH. The review identified this as a specific area for further research.

Arguably, to accurately determine the risk factors associated with PPH, a rigorous approach to blood loss measurement needs be implemented, and questions asked of staff caring for women during the intrapartum period exactly *how* the third stage of labour was conducted – rather than relying on estimation and self-reported definitions of practice. Furthermore, there are no recent Australian studies which have prospectively investigated the growing phenomenon of PPH and it is imperative that high-quality research is conducted to meet this need.

In 2015, 4672 (7.7%) women birthing in Queensland, Australia experienced a primary PPH [25]. At the time of this study the local area had a relatively high rate of women without known risk factors experiencing a severe PPH (6.8%) and it was important for the local health services to understand the reasons for this. So, within the context of rising incidence internationally and locally, this study aimed to prospectively explore variables associated with PPH in a cohort of women birthing vaginally in coastal Queensland, Australia, using the gravimetric method to measure blood loss.

## Methods

### Aim

This research study examined associations between independent risk factors and postpartum haemorrhage following a vaginal birth, prospectively in a cohort of pregnant women in a south-east coastal health care service, Queensland, Australia.

The study included the following objectives:

- Identify independent variables which are significantly associated with PPH;
- Determine which independent variables associated with PPH are modifiable;
- Describe accurate patterns of blood loss clearly through routine gravimetrically measured blood loss after vaginal birth;
- Describe specific components of current practice regarding modified-active third stage management.

### Design, setting and sample

A prospective cohort study was conducted within three sites:

1. Regional maternity unit (~ 2600 births per annum);
2. Rural maternity unit (~ 300 births per annum);
3. Eligible Private Practising Midwifery (EPPM) Service (with visiting rights at regional maternity unit and private home birth settings) (~ 50 births per annum)

The study population were pregnant and birthing women planning to birth within the three sites.

*Inclusion criteria*:

- Pregnant women booked in to birth within the regional and rural maternity units;
- Pregnant women booked in to birth under the care of the EPPM service (hospital or home);
- Planned vaginal birth.

*Exclusion criteria:*

- Pregnant women declining to participate in the study;
- Pregnant women planning to birth via lower section caesarean section (LSCS).

During 2014 a total of 2447 women birthed within the regional maternity unit with 1880 birthing vaginally (76.8%). An estimated 940 women were eligible to recruit during a six-month period.

All pregnant women planning a vaginal birth attending the routine midwife appointment within the study sites were informed about the study by the midwife conducting the consultation, with both a verbal summary and printed information sheet. The expectant woman was advised that the study was exploratory and did not involve any intervention and that perinatal care would continue as per usual. An opt-out consent process was approved, which is a method used in the recruitment of participants into research where information is provided to the potential participant regarding the research and their involvement and where their participation is presumed unless they take action to decline to participate. The participant information sheet and opt-out form were provided to all women during the consultation. The midwife then documented in the client health record that the woman had been informed about the study, as per local procedures. A total of three women chose to opt-out during the recruitment period.

### Data collection

Data were collected from October 2015 through until April, 2016. Informed by a review of the literature, data were prospectively collected, including identified characteristics of pregnant and birthing women. The primary outcome was a postpartum haemorrhage (gravimetric blood loss ≥500 ml). Within each study site in-service education was provided to all maternity staff working within the birthing suites to ensure gravimetric measurement of loss was attended consistently. Specialised scales were provided with a legend of ‘dry weight’ in grams of each absorbent pad used in the birthing suite, so that these could be deducted from blood soaked pads and a total loss calculated. This is consistent with other studies where grams are known to be equivalent to mls in weight [19, 26].

Data included: Maternal demographics; parity; previous pregnancy and birth history (including previous mode of birth and PPH); model of care; antenatal complications; current medical conditions; mode of birth; intrapartum complications; gravimetrically measured and estimated blood loss; third stage management practices; immediate postpartum complications (including PPH experienced in the post-natal ward following discharge from birth suite); haematological laboratory data.

Data were collected via a pre-designed data collection instrument, and entered directly into an electronic, password protected program (RedCAP) in a re-identifiable format by authors one and four. Data were coded and then transferred to a statistical software program for analyses.

### Data analysis

Descriptive statistics were calculated for demographic, intrapartum and birthing practice variables. Categorical variables are reported as counts with percentages. Continuous variables are reported as means with standard deviation where approximately normal, and median with interquartile range where skewed. A General Linear Model (GLM) was used for a multivariate analysis to examine the relationship between gravimetric blood loss (primary outcome) and demographic, birthing practice and intrapartum variables. A backwards stepwise procedure was used to remove variables with a significance greater than 0.10.

Demographic variables included were maternal age, body mass index at booking-in visit, smoking and previous births. Intrapartum variables were length of first, second and third stage of labour (minutes); perineal injury second degree or higher (yes/no); episiotomy (yes/no); labour onset induced (yes/no); complications arising during labour (yes/no); pharmacological pain relied used (yes/no); maternal position at birth (upright/non-upright/other); baby separated from mother in first hour after birth (yes/no). Birth practice variables included were placental birth management (active/mixed active/physiological); oxytocic administered after cord clamping (yes/no); signs of separation: cord lengthening (yes/no), gush of blood (yes/no), uterus risen (yes/no), maternal urge to push (yes/no), none observed (yes/no).

Some variables were excluded from analysis due to very small numbers (Australian Indigenous status, congenital abnormality, stillbirths). A log transformation was used on right skewed continuous variables (gravimetric blood loss; length of first, second and third stage of labour). The variable ‘The person who conducted the birth’ is likely confounded with ‘birth practices’ and was excluded from the multivariate analysis; this is examined separately with a Chi-squared test of independence.

SPSS version 24 was used for all analyses, with alpha less than 0.05 set as the level of significance.

## Results

### Participant characteristics

Over the seven-month data collection period there were 522 singleton births for whom complete data were collected within the participating health services. The mean age of mothers was 29 years, and 58% of mothers had had a previous birth (Table 1).

**Table 1:** Participant characteristics

	Sample	*N* (%)	Min, Max	Mean (SD)
Demography				
Maternal age (years)	522		14, 44	29.10 (5.56)
Body Mass Index (BMI) at antenatal ‘booking-in’ visit	514		15, 47	24.25 (5.42)
Birth weight, g	505		1690, 4820	3487 (473)
< 2500		5 (1)		
2500–3999		439 (84.1)		
> 4000		61 (11.7)		
Baby’s gestation at birth	521		33.6, 42.3	39.6 (1.4)
- < 32 weeks		0 (0)		
- 32–36 weeks		20 (3.8)		
- ≥ 37 weeks		501 (96.2)		
Marital Status	522			
- Married/defacto		446 (85.4)		
- Never married		68 (13.0)		
- Widowed, divorced, separated		8 (1.6)		
Aboriginal and/or Torres Strait Islander (yes)	522	18 (3.5)		
Any smoking during pregnancy (yes)	518	49 (9.5)		
Previous pregnancy (yes)	522	358 (69.1)		
Previous birth (yes)	522	303 (58.0)		
Previous Births				
Total number of previous pregnancies (excluding nulliparous women)	*354*		1, 11	2.08 (1.5)
Previous birth type^a^				
- Spontaneous Vaginal Birth	*303*	247 (81.5)		
- Instrumental Vaginal Birth	*303*	44 (14.5)		
- Caesarean Birth	*303*	19 (6.3)		
Previous PPH (yes)				
- No	*271*	241 (88.9)		
- Yes	*271*	14 (5.2)		
- Unknown	*271*	16 (5.9)		
Current Birth				
Received antenatal care (yes)	522	516 (99.6)		
Primary mode of care	518			
- Midwife (public)		297 (57.3)		
- Midwife (private)		32 (6.2)		
- Obstetric clinic (public)		16 (3.1)		
- GP shared clinic		160 (30.9)		
- Other		13 (2.5)		
Place of birth	522			
- Regional maternity unit		466 (89.3)		
- Rural maternity unit		35 (6.7)		
- Regional maternity unit (EPPM care)		13 (2.5)		
- Home birth (EPPM care)		8 (1.5)		
Placental Birth Management	522			
- Active		291 (55.7)		
- Modified/mixed active		191 (36.6)		
- Physiological		40 (7.7)		

^a^Women may have had more than one previous birth

Table 2 presents the data pertaining to the intrapartum period.

**Table 2:** Intrapartum factors

Variable	*N*	*n*	%	Median (IQR)
Duration of labour				
Length of first stage	520			255 (269)
Length of second stage	522			21 (38)
Length of third stage	522			11 (9)
Perineal Injury	522			
Perineal graze		28	5.4	
1st degree perineal injury		69	13.2	
2nd degree perineal injury		195	37.4	
3rd degree perineal injury		21	4	
4th degree perineal injury		1	0.2	
Labial tear/s		60	11.5	
Episiotomy		60	11.5	
Onset of labour induced^a^	522	149	28.5	
Induction methods used - PGE		92	17.6	
Induction methods used - Cooks catheter		8	1.5	
Induction methods used - ARM		97	18.6	
Induction methods used - Syntocinon		54	10.3	
Labour augmented by an ARM		168	32.2	
Labour augmented with syntocinon		69	13.2	
Pain Relief	522			
Pharmacological pain relief - epidural		125	23.9	
Pharmacological pain relief - spinal		1	0.2	
Pharmacological pain relief - nitrous		319	61.1	
Pharmacological pain relief - morphine		57	10.9	
Pharmacological pain relief - pethidine		4	0.8	
Pharmacological pain relief - caudal		1	0.2	
Complications	522			
Complications arising during labour		185	35.4	
Complications during labour - Failure to progress		6	1.1	
Complications during labour - Precipitate labour		13	2.5	
Complications during labour - Shoulder dystocia		5	1	
Complications during labour - Failed Vacuum		5	1	
Complications during labour - Maternal temperature		4	0.8	
Complications during labour - Meconium Liquor		69	13.2	
Complications during labour - Fetal distress		42	8	
Complications during labour - Gestational hypertension		6	1.1	
Complications during labour - Pre-eclampsia		5	1	
Complications during labour - Abruption		4	0.8	
Complications during labour - Intrapartum Haemorrhage		3	0.6	
Complications during labour - Cord entanglement		3	0.6	
Complications during labour - Prolonged second stage		46	8.8	
Complications during labour - HELLP		0	0	
Complications during labour - Eclampsia		0	0	
Complications during labour - Cord prolapse		0	0	
Complications during labour - Failure forceps		0	0	
Maternal position at birth	515			
Upright (all fours, standing, squatting, kneeling, birth stool, lateral)		183	35.5	
Non-upright (semi-recumbent, supine, lithotomy)		278	54	
Other or unknown		54	10.5	
Placental position	520			
Anterior		265	51	
Posterior		214	41.2	
Fundal		20	3.8	
Low-lying		3	0.6	
Unknown		18	3.5	
Presentation at birth	522			
Vertex		521	99.8	
Breech		1	0.2	
Who conducted the birth?	522			
Midwife (registered midwife, student midwife, or midwife supervised)		407	78	
Doctor (JHO, obstetrician, JHO)		115	22	
Baby separated from mother within first hour after birth	522	75	14.4	
Congenital abnormality at birth	522	3	0.6	
Born alive or still	522	519	99.4	

^a^total of induction methods is greater than “yes” for onset of labour induced as more than one method may have been used

### Third stage management practices

The majority of participants had the third stage of labour actively or modified actively managed (active 291; 55.7%; modified active 191; 36.6%; expectant 40; 7.7%). The key reason for selecting this approach was most commonly cited as maternal preference (258; 49.6%), followed by presence of risk factors for PPH (146; 28.1%). Further exploration of third stage management practices within this study have been reported elsewhere.

### Gravimetric blood loss

A total of 292 women in the study had a non-pathological blood loss of 0-499 ml, with 128 experiencing a PPH, and an additional 31 experiencing a severe PPH, gravimetrically measured.

The primary outcome in this study was gravimetric blood loss, which was strongly correlated with the estimated blood loss (*r* = 0.88; *p* < 0.001). On average, the estimated blood loss was lower than the gravimetric blood loss, about 78% of the measured value. However, the majority of estimations were relatively accurate, 76% of estimated values were within 100 ml of measured values (either side).

**Fig. 1:**
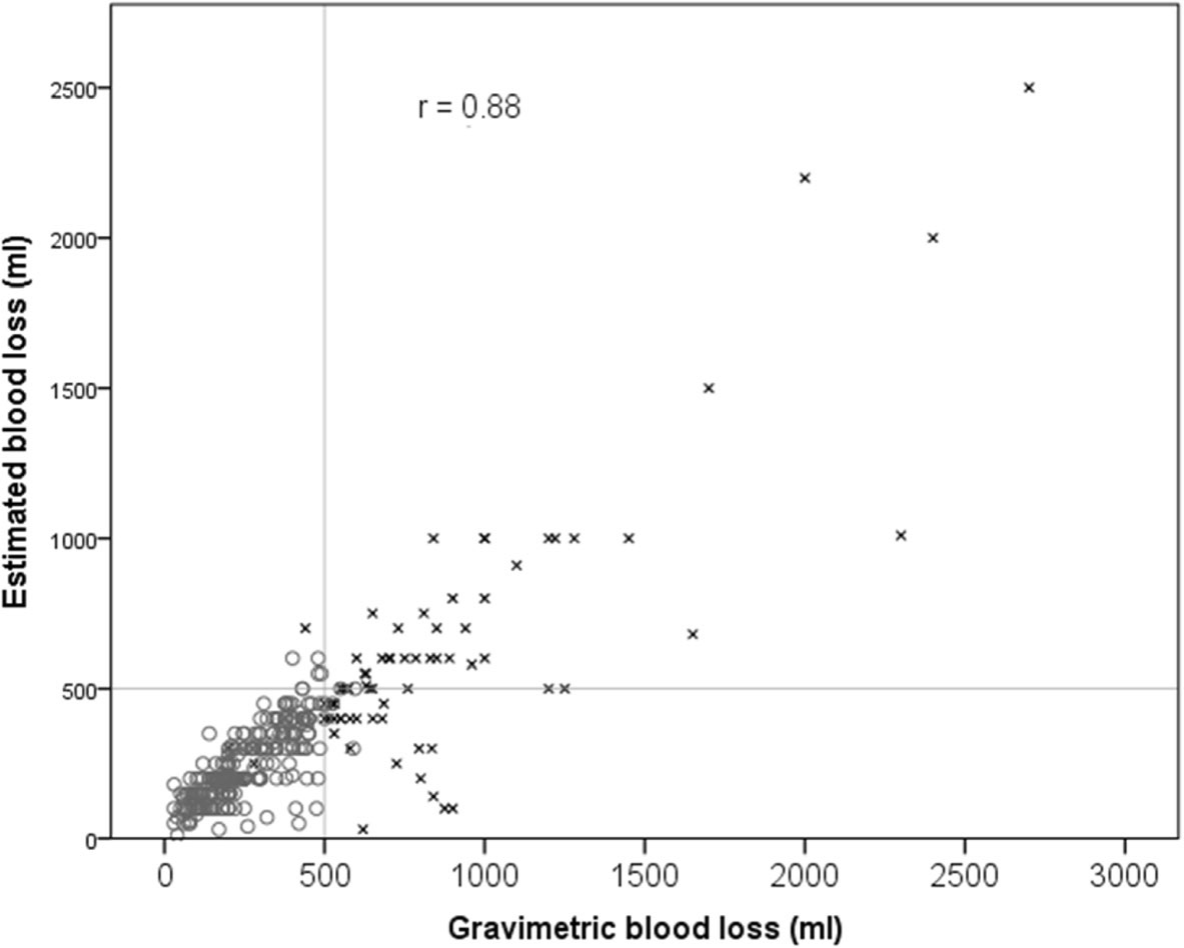
Estimate and gravimetric blood loss recorded at PPH

**Fig. 2:**
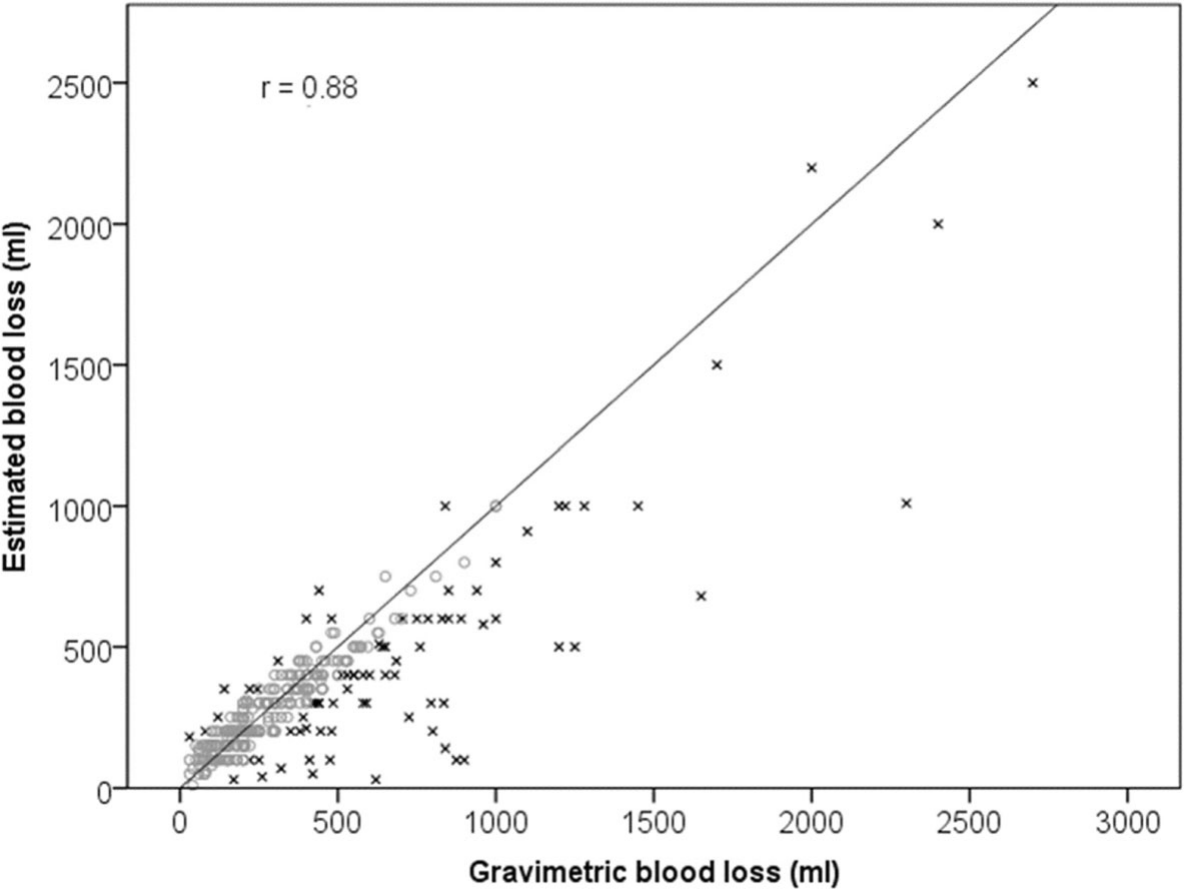
Estimated and gravimetric blood loss with a reference line where the two values are equal. [All points below the line have been underestimated, all points above the line have been overestimated. x indicates estimated blood loss is greater than a 100 ml different from gravimetric blood loss]

Current definitions suggest that a blood loss of greater than 500 ml is regarded as a post-partum haemorrhage. Out of 476 births with valid estimated blood loss recorded, 93 (20%) recorded an estimated blood loss of 500 ml or more, with the majority accurately diagnosed as a PPH (64,;78%). Similarly, out of 451 births with valid gravimetric blood loss recorded, 159 (35%) recorded a gravimetric blood loss of 500 ml or more most of these (111; 70%) were accurately diagnosed as a PPH (see Figs. 1 and 2).

Table 3 presents the multivariate analysis of gravimetric blood loss.

**Table 3:** Multivariate analysis with the dependent variable as the log transform of gravimetric blood loss

	Full model		Reduced model	
	*Effect size (η*_*p*_^*2*^ *)*	Sig	*Effect size (η*_*p*_^*2*^ *)*	Sig
Intercept	.060	.000	0.232	0.000
Maternal Age	.003	.331		
BMI	.001	.487		
Baby Birth Weight	.017	.013	0.018	0.009
Gestation	.006	.132		
(Ln) Length First Stage	.006	.163	0.009	0.070
(Ln) Length Second Stage	.001	.537		
(Ln) Length Third Stage	.000	.768		
Any Smoking (yes/no)	.002	.395		
Previous Birth (yes/no)	.000	.841		
Perineal Injury 2nd-4th grade (yes/no)	.054	.000	0.050	0.000
Episiotomy (yes/no)	.001	.510		
Labour onset induced (yes/no)	.004	.210		
Labour complications (yes/no)	.016	.017	0.018	0.008
Pharmacological Pain Relief (yes/no)	.001	.600		
Maternal position (upright, non-upright, other)	.008	.240		
Baby separated from mother within first hour of birth (yes/no)	.008	.101	0.010	0.046
Placental birth management (active/mixed active/physiological)	.031	.004	0.033	0.002
Oxytocic after cord clamping (yes/no)	.015	.066	0.013	0.086
Signs of separation: cord lengthening (yes/no)	.002	.370		
Signs of separation: gush of blood or clot (yes/no)	.044	.000	0.044	0.000
Signs of separation: uterus risen above umbilicus (yes/no)	.004	.208		
Signs of separation: maternal urge to push (yes/no)	.003	.274		
Adjusted R Squared	0.165		0.167	

**Fig. 3:**
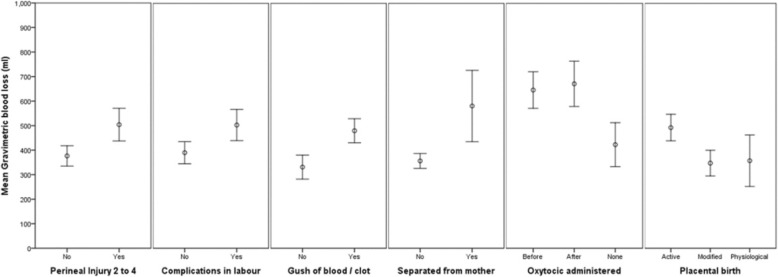
Means and confidence intervals for significant variables

Variables were removed in a stepwise backwards procedure until no variables had a significance greater than 0.10. [Order of variables removed: previous births; ln third stage: pharm pain relief; Signs of Separation - cord lengthening; ln second stage: BMI; Signs of Separation uterus risen; maternal position; episiotomy; Signs of Separation - maternal urge push; any smoking; gestation; labour onset; maternal age]. Factors associated with higher gravimetric blood loss were perineal injury (requiring suturing) (*p* < 0.000); complications arising during labour (*p* < 0.008); separation of mother and baby during the first hour after birth (*p* < 0.046); a gush of blood observed as a sign of placental separation prior to the placenta being delivered (p < 0.000); oxytocic administration (before or after cord clamping compared with no oxytocic administered) (*p* < 0.086) and an active third stage management (p < 0.000) – see Fig. 3. Increasing birth weight (*p* < 0.009) and length of first stage of labour (*p* < 0.070) were also associated with higher gravimetric blood loss. All effect sizes are small to medium.

## Discussion

Postpartum haemorrhage rates continue to rise throughout industrialised countries, and whilst key risk factors have been identified [9, 14], the rate at which PPH has increased is not explained by the contemporaneous changes seen in known risk factors. This study aimed to identify if variables such as third-stage management practices and other specific factors which may be associated with PPH were contributing to the higher than average PPH rate in the study location within Queensland, Australia. The overall rate of PPH (defined as blood loss ≥500 ml following vaginal birth) was 28.1% (*n* = 128), and severe PPH (defined as blood loss ≥1000 ml following vaginal birth) was 6.8% (*n* = 31), which is higher than reported rates in other Queensland hospitals [25]. Several variables were significantly associated with PPH in this cohort, including high neonatal body weight, perineal injury (requiring suturing), complications arising during labour, separation of mother and baby during the first hour after birth, and a gush of blood observed as a sign of placental separation prior to the placenta being delivered. Nulliparity, labour induction and augmentation, and synthetic use of syntocinon did not arise as significant risk factors in this study, contrary to other literature [14, 17].

Birth weight is reported consistently in the literature as a predictive factor associated with postpartum haemorrhage [7, 17, 27] and subsequent postpartum anaemia [27]; with a positive correlation found between increasing risk of PPH and increasing birth weight. Mean birth weight for sample infants was 3487 (± 448) grams with fetal macrosomia (≥4000 g) contributing 11.7% (*n* = 61) to this sample; consistent with figures reported for infants born 4000 g or more in Queensland (11%) during 2015 [25]. In this sample, increasing birth weight remained a significant factor associated with PPH in the GLM model and the reduced linear model, consistent with previous reports [14, 27]. However, the average birth weight of neonates within Queensland has not increased significantly over the past 15 years (3413 g in 2000 compared with 3407 g in 2015), thus not explaining the increasing trend for PPH within the local area. Perineal injury (including episiotomy) is also a known contributing factor to increased blood loss after birth [9, 27], and was supported as a contributing factor to PPH in this study.

Complications arising during labour and birth occurred in 185 women (35.4%) of the cohort, and were significantly associated with PPH (*p* = 0.012). Complications were defined in Table 2 and were aggregated for the GLM model, due to low numbers amongst specific complications. The rate of any complications arising during labour and birth has steadily risen over the past 15 years throughout Queensland, with 27.7% of women birthing without complication in 2000, decreasing to 21.3% in 2015. This is likely to be associated with the increasing PPH rates.

For maternity health professionals this finding is relevant, in that whilst women may initially present in labour as ‘low-risk’ without known risk factors, complications may arise during the labour and birth which should be considered and inform third-stage management practices. Specifically, an actively managed third-stage of labour should be recommended [6], within a shared decision-making framework with the woman and her support people, to enhance safety and reduction of risk of PPH [2]. Ideally this conversation should occur prior to labour and birth and is best situated in a continuity of care model [28, 29].

Conversely women in spontaneous labour, without complications arising during labour and birth have demonstrated less likelihood of PPH with physiological management of the third stage of labour [30–32]. Whilst the mechanisms regarding this are not well understood, it has been hypothesized that interruption of endogenous oxytocin at any stage during labour and birth (including placental delivery) may effect optimal release and uptake of oxytocin by the uterine muscle [33], essential in reducing blood loss after birth. One such interruption to the physiological process is separation of mother and baby in the first hour after birth, which was significantly associated with PPH in our study. This confirms findings from Saxton and colleagues study [34] which found women who did not have uninterrupted skin to skin or initiate breastfeeding within the first hour were twice as likely to experience a PPH after adjustment for covariates (aOR 0.55, 95% CI 0.41–0.72, *p* < 0.001). Whist Saxton and colleagues study was limited by the retrospective, estimated primary outcome measure, our study substantiates their findings with a prospective, gravimetrically measure primary outcome measure.

Debate continues within the wider literature on inaccuracy of postpartum blood loss estimation, with experts suggesting that improving assessment of blood loss after birth is a critical step in early detection, and thus early management, of PPH [35]. Hancock and colleagues [36] conducted a comprehensive integrative review evaluating the various methods of assessing maternal blood loss during childbirth. Key findings from this review were that health professionals were highly inaccurate in the estimation of blood loss as volume and that training afforded some short-term improvement, but this was not sustained and did not eventuate in improvements to clinical outcomes [36]. Other aspects of assessment were identified by health professionals which aided their clinical decision making, including speed of blood flow and the woman’s clinical condition, which have received less attention in the literature and academic discussion. Other novel approaches to aid accurate blood loss measurement are also being more widely utilized, such as under-buttocks drapes – which are effective if the mother is birthing supine or semi-recumbent on a bed [37], but challenging in other positions.

Within our study, midwives were asked to identify which signs of placental separation were observed. Observation of a ‘gush of blood,’ which is well recognised as a sign of placental separation [1], was significantly associated with a PPH following vaginal birth in our study. Given this unique finding could be consistent with Hancock and colleagues’ [36] argument that not only is blood loss volume important to assess, but also the type and speed at which blood loss occurs, further scientific exploration is warranted. Earlier recognition and appropriate treatment of abnormal blood loss has potential for both human and fiscal resource savings [38].

As the midwives and obstetric doctors were requested to both estimate and then gravimetrically measure the blood loss of women included in this study, interesting comparisons were made. The primary outcome in this study was gravimetric blood loss, which was strongly correlated with the estimated blood loss (*r* = 0.88; *p* < 0.001). On average, the estimated blood loss was lower than the gravimetric blood loss; about 78% of the measured value. However, the majority of estimations were relatively accurate; 76% of estimated values were within 100 ml of measured values (either side). This too is an important finding as the sample size of this study was considerably larger than previous simulated example studies [22, 23] and Al Khadri and colleagues’ prospective study [19]. More accurate diagnosis of emerging PPH, and prompt and effective management may explain the comparatively high rate of non-severe PPH (blood loss of 500-999 ml) (128; 28.1%), in the study sites, and reflects positively on the health professionals’ involved ability to identify early and effectively manage PPH following vaginal birth. Natrella and colleagues [39] found that blood loss estimation becomes more inaccurate as the blood loss volume increases. Routine gravimetric measurement has been introduced into practice without evidence of its effectiveness at improving the management of PPH. It can be argued that the act of collecting and measuring blood loss may distract the mother and disrupt the release of endogenous oxytocin [40]; in addition to redirecting the health professional’s focus from the woman. In this study health professionals’ estimations of blood loss were relatively accurate, and discrepancies were not clinically significant. These findings support the use of gravimetric measurement in response to a variance in blood loss rather than as a routine practice.

### Limitations

It was beyond the scope of this study to explore factors associated with PPH after caesearean birth. Known PPH risk factors, such as caesarean birth, multiple pregnancies, placenta previa and transverse lie exist within the literature, but were not included in this study.

## Conclusions

This study identified several variables associated with PPH that support the findings of previous studies. High neonatal weight, perineal injury, complications during labour, and separation of mother and baby in the first hour after birth were risk factors for PPH. In contrast to previous study findings, nulliparity, labour induction and augmentation, and use of syntocinon were not independently associated with PPH in this study. This study also found that a health professional’s estimation of blood loss was relatively accurate in comparison to gravimetric assessment. This finding raises questions about routine gravimetric assessment of blood loss following uncomplicated births. The observation of a ‘gush of blood’ prior to the birth of the placenta was a significant risk factor for PPH in this study. This unique finding suggests a need for further research into the type and speed of blood loss associated with PPH.

## Abbreviations

GLM: General Linear Model
PPH: Postpartum haemorrhage
